# Yield Stability Analysis of Maize (*Zea mays* L.) Hybrids Using Parametric and AMMI Methods

**DOI:** 10.1155/2021/5576691

**Published:** 2021-03-25

**Authors:** Seyed Habib Shojaei, Khodadad Mostafavi, Ali Omrani, Saeed Omrani, Seyed Mohammad Nasir Mousavi, Árpád Illés, Csaba Bojtor, Janos Nagy

**Affiliations:** ^1^Department of Agronomy and Plant Breeding, Science and Research Branch, Islamic Azad University, Tehran, Iran; ^2^Department of Agronomy and Plant Breeding, Karaj Branch, Islamic Azad University, Karaj, Iran; ^3^Crop and Horticultural Science Research Department, Ardabil Agricultural and Natural Resources Research and Education Center, AREEO, Moghan, Iran; ^4^Plant Breeding and Genetics, Department of Agronomy and Plant Breeding, Isfahan University of Technology, Isfahan 84156-83111, Iran; ^5^Institute of Land Utilization, Technology and Regional Development, Faculty of Agricultural and Food Sciences and Environmental Management, University of Debrecen, Debrecen, Hungary

## Abstract

The present study investigated the stability and adaptability of maize (*Zea mays* L.) hybrids. In this study, 12 maize hybrids were planted and examined considering the grain yield. The experiment was arranged in a randomized complete block design (RCBD) with three replications in four research stations in Iran during two crop years. The combined analysis of variance showed that genotype-environment interactions were significant at one percent probability level. The grain yield can stabilize, and hybrids with specific adaptability are recommended to each environment. Hybrids with specific adaptability can be recommended to all types of the environment. Means comparison yield of the genotypes identified DC370 as a high-yield genotype. Regarding AMMI analysis, genotype × environment interactions (GEIs) and two first components were found significant. The SC647 genotype was identified as the most stable genotype. Regarding the stability parameters, SC647 and KSC705 genotypes were selected as the most stable genotypes. From AMMI1 and AMMI2 graphs, the SC647 genotype was identified as the most stable genotype compared with other hybrids.

## 1. Introduction

Maize is one of the various crucial annual cereal crops, accounting for more than 188 Mha of croplands worldwide and contributing nearly 50% (1.171 Mt) of global food production [1]. The production and trade of maize (*Zea mays* L.) targeted at animal husbandry. However, maize has been a leading and integral staple food for humans. Considering the importance of maize and its cultivation potential worldwide and in Iran, this crop's yield sustainability was studied in previous research. Genotype × environment interaction (GEI) is responsible for genotype sustainability characteristics [2–4]. One of the usual exacting effects in plant breeding development is to dissect the genotype × environment (GE) interaction correctly. In most trails, the *G* × *E* interaction was witnessed and then modeled statistically and elucidated. Genotype × environment interaction adjusts the reasonable grain yield of genotypes in diverse environments and makes it hard to select the better ones [5]. Multilocation and multiyear trials can help identify superior and sustainable genotypes [6]. It can also help to identify different environments through the differences between genotypes with minimal replicates. Environments with better yield can distinguish superior genotypes in target environments [7]. Identifying high-yielding and sustainable genotypes in diverse environments has been a constant challenge for plant breeders worldwide. Features of sustainable genotypes made possible by successive genotype × environment interactions (GEI). Since the environment very much influences genotypes, locations, and years, performance related to the stability of genotypes is a prerequisite and must carry out in different environments over different years in a given location [8]. Plant breeders are evaluating genotypes in multienvironment trials (MET), including in favorable and unfavorable environmental conditions on the genotype × environment interaction (GEI). MET variance analysis provides reasonable estimates of the critical effects of genotype (*G*) and environment (*E*) and genotype × environment interaction (GEI) in which the effects of *G* and GEI mainly lead to cultivar evaluation. The value of observed genetic variance varies from one environment to another due to interaction effect, and it tends to improve in superior environments higher than unfavourable environments [9]. The genotype-environment interaction (GEI) for quantitative traits such as grain yield can limit the selection of superior genotypes to develop modified cultivars [10]. To calculate GEI, breeders evaluate genotypes in various environments to identify high-yielding and stable genotypes. Genotypes with no GEI are considered stable genotypes [11]. Yield stability is one of the most desirable traits of a genotype that allows it to identify as a cultivar. The construction of a large-scale facility is required to study genotypes, referring to as specific adaptability. To achieve maximum production, it is essential to develop hybrid varieties that best fit given target environments and have specific adaptability. Additive main effects and multiplicative interaction analysis (AMMI) allow for a large set of technical interpretations, and they were used more commonly to evaluate the genotype-environment interactions. The additive main effects and multiplicative interaction (AMMI) model addresses the limitations of ANOVA and PCA. The AMMI model effectively explains the GEI patterns. The AMMI method is used for three primary purposes for stability. The first is that the model involves variance analysis and principal component analysis. Second, AMMI clarify GEI and summarize *G* and *E* patterns and relationships, and the third use is the accuracy of yield estimates [12]. The AMMI model is more suitable and simplifies genotypes' instantaneous choice for stability. The model helps establish the relationship of genotypes, environment, and their interaction [13, 14]. AMMI stability value (ASV), the distance of the coordinates of each genotype from the origin of the biplot coordinate diagram of the two principal components of the interaction, is based on the scores of the first and second for interaction principal component axis (IPCA) model for each genotype [15]. Genotypes with the lowest ASV are identified by their shortest projection from the biplot origin and considered the most stable [16]. Farshadfar et al [17] used the yield stability index (YSI), which combined high yield performance with stability. The YSI based on the sum of the ranking due to ASV scores and yield or performance ranking. Lower YSI indicates genotypes that combine high yield or performance with stability [18, 19].

## 2. Materials and Methods

### 2.1. Data Source

In this study, 12 maize hybrids were planted and examined considering the grain yield. This experiment was arranged in a randomized complete block design (RCBD) with three replications at four research stations in Iran during two crop years. The names and characteristics of the research stations are presented in Table 1. The specifications of the hybrids used in the experiment are presented in Table 2.

### 2.2. Statistical Analysis

The AMMI model according to Farshadfar et al. [17] is presented as follows:

(1) $$\begin{array}{c} Y_{ij}=\mu +g_{i}+e_{j}+\sum_{k=1}^{n}\lambda_{k}\alpha_{ik}\gamma_{jk}+e_{ij}, \end{array}$$

where *Y*_*ij*_ is the yield of the *i* genotype in the *j* environment; *g*_*i*_ is the mean of the *i* genotype; *λ*_*k*_ is the square root of the eigenvalue of the PCA axis *k*; *α*_*ij*_ and *γ*_*jk*_ are the principal component scores for PCA axis *k* of the I genotype and the *j* environments; and *e*_*ij*_ is the residual.

### 2.3. Parametric Measures

Shahbazi [20] and Liu et al. [21] proposed and had used the below formulas to study the stability. Wricke's ecovalence, which is simpler to calculate, is for the genotype *i*:

(2) $$\begin{array}{c} W_{i}^{2}=\sum {\left(x_{ij}-{\bar{x}}_{i.}-{\bar{x}}_{.j}+{\bar{x}}_{..}\right)}^{2}, \end{array}$$

where *X*_*ij*_ is the observed yield response (averaged across experiment replicates), *X*_*i*_. and *X*_*j*_ correspond to the previous notations, and *X* is the grand mean.

Greatest stability is when *W*_*i*_^2^ = 0.

The stability parameter is called stability variance (*δ*_*i*_^2^), which is obtained using the following equation:

(3) $$\begin{array}{c} \delta_{i}^{2}=\left[\frac{P}{\left(p-2\right)\left(q-1\right)}\right]\times {\left(x_{ij}-{\bar{x}}_{i.}-{\bar{x}}_{.j}+{\bar{x}}_{..}\right)}^{2}-\frac{\text{SSGE}}{\left(p-1\right)\left(p-2\right)\left(q-1\right)}. \end{array}$$

In the above equation, the sum of squares of the genotype-environment interaction is obtained as follows:

(4) $$\begin{array}{c} \text{SSGE}=\sum_{i}Wi=\sum_{i}^{p}\sum_{j}^{q}{\left(x_{ij}-{\bar{x}}_{i.}-{\bar{x}}_{.j}+{\bar{x}}_{..}\right)}^{2}. \end{array}$$

According to stability variance (*δ*_*i*_^2^), a stable genotype has minimal stability variance.

Stability was also measured by combining the coefficient of variation (CVi) and mean yield. Genotypes with low CVs and high average yields were regarded as most desirable.

Using joint regression analysis to study GE interaction, genotype effects and interaction effects within unique environments are linked to environmental impacts. The interaction sum of squares is separated into two sections: one section expresses the heterogeneity of linear regression coefficients (*b*_*i*_), and the second section expresses the pooled differences from unique regression lines (*S*_di_^2^).

Eberhart and Russell [22] suggested an evaluation of hybrid responses to environmental variations applying a linear regression coefficient (*b*_*i*_) plus the variance of the regression differences (*S*_di_^2^):

(5) $$\begin{array}{c} b_{i}=1+\frac{\sum_{i}\left(X_{ij}-{\bar{X}}_{i.}-{\bar{X}}_{.j}+{\bar{X}}_{..}\right)\left({\bar{X}}_{.j}-{\bar{X}}_{..}\right)}{\sum_{j}{\left({\bar{X}}_{.j}-{\bar{X}}_{..}\right)}^{2}},\\ S_{\text{di}}^{2}=\frac{1}{E-2}\left[\underset{i}{\sum }\left(X_{ij}-{\bar{X}}_{i.}-{\bar{X}}_{.j}+{\bar{X}}_{..}\right)-{\left(b_{i}-1\right)}^{2}\underset{i}{\sum }{\left({\bar{X}}_{.j}-{\bar{X}}_{..}\right)}^{2}\right]. \end{array}$$

*X*_*ij*_ is the grain yield of genotype *i* in environment *j*; *X*_*i*_. is the average yield of hybrid *i* and *X*_.*j*_ is the average performance of the environment *j*; *X*. is the grand mean, and *E* is the number of environments.

The environmental variance is one of the significant stability measures of the static stability concept and is calculated for each genotype across test environments. A genotype with minimum variance under different environments was considered to be stable. This measure was calculated as follows:

(6) $$\begin{array}{c} S_{Xi}^{2}=\frac{\sum {\left(X_{ij}-{\bar{X}}_{i.}\right)}^{2}}{\left(E-1\right)}, \end{array}$$

where *X*_*ij*_ is the grain yield of genotype *i* in environment *j*, *X*_*i*._ is the mean yield of genotype *i*, and *E* is the number of environments.

The AMMI stability value was calculated as previously described by Purchase et al [15] and used by Abate [23] and Adjebeng-Danquah et al. [24]:

(7) $$\begin{array}{c} \text{ASV}=\sqrt{{\left[\frac{{\text{IPCA}1}_{\text{SQ}}}{{\text{PCA}2}_{\text{SQ}}}\left({\text{PCA}1}_{\text{score}}\right)\right]}^{2}+{\left({\text{IPCA}2}_{\text{score}}\right)}^{2}}, \end{array}$$

where IPCA1_SQ_/PCA2_SQ_ is the weight obtained from separating the IPCA1 sum of squares by the IPCA2 sum of squares. The greater the total amount of IPCA, the higher the adaptability of a particular variety for a particular environment. Conversely, lower ASV scores indicate more excellent stability in different environments.

The performance stability index calculated applying the sum of the ranking based on performance and ranking based on the AMMI stability amount.

(8) $$\begin{array}{c} \text{YSI}=\text{RASV}+\text{RY}. \end{array}$$

RASV is the genotypes' rank based on the AMMI stability value, and RY is the rank of the genotypes based on yield across environments [12, 25].

## 3. Results

A combine analysis of variance showed that environmental effect, genotype effect, and genotype-environment interaction were significant at one percent probability level. Significance of the effect of environments indicated that environments differed in yield of genotypes, and the significance of GEI showed that the yield of genotypes varied in different environments (Table 3).

Considering Duncan's mean comparison in 1% probability level on these data and yield of genotypes, KSC707 ranked the highest and KSC704 and SC301 ranked the lowest (Table 2).

Kandus et al. [26] stated that the best model is when only the first two principal components are significant, and the other components showed little variation. The first and second principal components are representing the genotypes and environments, respectively, as can be seen in Table 4.

The environments with the first significant interactions are suitable for identifying and screening genotypes. In this study, the E2 environment was selected as the most suitable environment for screening the genotypes. SC647 genotype with a relatively high yield showed the lowest first principal component of interaction, so it is highly stable. It can be used as a desirable genotype for planting in different regions and years (Table 4). According to the environmental variance method, a genotype with the lowest environmental variance has been selected as the most desirable hybrid; SC604 and KSC703 genotypes had less environmental variance than the rest of the genotypes and hence were considered the most desirable genotypes in this method. SC302 and KSC400 genotypes having the highest environmental variance were identified as the most unstable hybrids. According to the environmental coefficient of variation (CVi), a stable genotype has the least value of this parameter. KSC707 and SC604 genotypes had the highest values of mean rank of parameters, and hence, they were identified as the most stable hybrids in this method. Also, KSC400 and SC302 genotypes having the highest environmental variation coefficient were identified as the most unstable hybrids. These hybrid had a smaller percentage in genotype and environment interaction, according to Wricke's ecovalence and Shukla's stability variance parameters. The Based on the results of these two methods, KSC705, KSC706, and KSC704 genotypes were identified as the most stable hybrids. Also, KSC703, KSC707, and DC370 genotypes were identified as the most unstable genotypes because of the high level of these two parameters. The high value of regression (*b*_*i*_ > 1) indicates that the variety is more responsive for the input-rich environment, while the low value of regression (*b*_*i*_ < 1) is an indication that the variety may be adopted in a low environment. Thus, the KSC400 genotype is more responsive for the input-rich environment, and the KSC703 genotype is adopted in a low environment. Considering Eberhart and Russell's regression coefficient (*S*_di_^2^), any genotype with the least value was more stable. Consequently, KSC704 and SC647 genotypes having the lowest mean yield parameter were recognized as the most stable hybrids. SC302 and SC301 genotypes having the highest mean yield parameter were identified as the most unstable genotypes. Based on the detection coefficient method, a stable genotype benefits from this highest statistic value, according to which KSC705, KSC706, KSC704, and SC647 hybrids with the highest parameters were identified as the most stable genotypes. The KSC707 genotype was identified as one of the most unstable hybrids due to its high statistic level. Although the methods mentioned above have similarities in identifying the stable genotypes, some differences indicate that the above parameters' results are not wholly consistent. In the environmental variance method, KSC703 and SC604 hybrids were selected as the most stable genotypes; in the coefficient of variation, SC604 and KSC707 hybrids; in the genotypic variance-based method, KSC705, KSC706, and KSC704 hybrids; in Eberhart and Russell's regression coefficient method, KSC704 and SC647; and in detection coefficient method; KSC706, KSC704, and SC647 hybrids were selected as the most stable genotypes, which reveal the differences in the hybrid selection. KSC400 and SC302 genotypes were selected as the most unstable genotypes in identifying the most unstable hybrids based on environmental coefficient of variation; based on variance methods, KSC703, DC370, and KSC707 genotypes; based on Eberhart and Russell's regression coefficient method, SC302 and SC301 genotypes; and based on detection coefficient, KSC707 were selected as the most unstable hybrids. The univariate analysis was performed on the data of four regions in the second crop year. A genotype with the least environmental variation was selected as the most desirable hybrid. The SC647 genotype showed less environmental variation than the rest of the genotypes and hence was recognized as the most desirable genotype of this method. KSC703 and KSC705 genotypes identified as the most unstable hybrids because of having the highest environmental variance. According to the environmental coefficient of variation (CVi), a stable genotype showed the lowest value of this parameter, and accordingly, SC647 and KSC707 genotypes had the lowest value of this parameter and hence were identified as the most stable hybrids of this method. Also, KSC705, KSC706, and KSC704 genotypes were identified as the most unstable hybrids because of having the highest environmental variation coefficient. According to GEI and environmental variance methods, including Wricke's ecovalence and Shukla's stability variance parameters, the lower these two statistics, the higher the stability of the hybrids. Based on the results of these two methods, SC647, SC302, and SC301 genotypes were identified as the most stable hybrids because of having the lowest values in both statistics. Also, KSC706 and KSC704 genotypes were identified as the most unstable genotypes because of having the highest values in both statistics. According to the *b*_*i*_ parameter, DC370, SC604, KSC707, and KSC705 genotypes are more responsive for input-rich environments, and the KSC706 genotype adopted in a low environment. Regarding Eberhart and Russell regression coefficient, any genotype with the least value of this parameter is more stable. Consequently, SC302 and SC647 genotypes with the least value of this parameter identified as the most stable hybrids. In addition, KSC703, KSC704, and KSC706 were identified as the most unstable genotypes because of their high value of this parameter. Based on the detection coefficient method, a stable genotype showed the highest value, and so KSC705, SC647, and SC301 hybrids were identified as the most stable genotypes. KSC400, KSC706, and DC370 genotypes were identified as the most unstable hybrids due to their high value. The univariate analysis was performed on the data of four regions in two crop years. The genotypes with the lowest value were selected as the most desirable hybrids considering the environmental variance, based on which KSC707, DC370, SC647, and SC604 genotypes showed less environmental variance than the rest of the genotypes, and hence they were identified as the most desirable genotypes of this method. The KSC705 genotype was identified as the most unstable hybrid since it showed the highest environmental variance. According to the environmental coefficient of variation (CVi), a stable genotype valued the least, and based on which the KSC707 genotype showed the lowest value of the parameter and hence was identified as the most stable hybrid of this method. Also, KSC705, KSC400, KSC706, and KSC704 genotypes were identified as the most unstable hybrids with the highest environmental coefficient of variation. According to GEI and environmental variance methods, including Wricke's ecovalence and Shukla's stability variance parameters, the lower these statistics in the genotypes, the higher the stability of the hybrids; and based on the obtained results, KSC260, KSC705, and SC647 genotypes were identified as the most stable hybrids due to their lowest values in both statistics. Also, KSC703, KSC706, KSC707, and DC370 genotypes were identified as the most unstable genotypes due to their highest levels of these two statistics. According to the *b*_*i*_ parameter, KSC705 is more responsive for an input-rich environment, and the DC370 genotype was adopted in a low environment. According to Eberhart and Russell regression coefficient, any genotype with the least value of this parameter is more stable. Consequently, KSC260, KSC707, and SC647 genotypes had the least value of this parameter, and they were identified as the most stable hybrids. KSC706 was identified as the most unstable genotype because it showed the highest levels of this parameter. The genotype was identified as stable when it showed the highest value of this parameter considering the detection coefficient (Table 5).

## 4. Discussion

**Figure 1:**
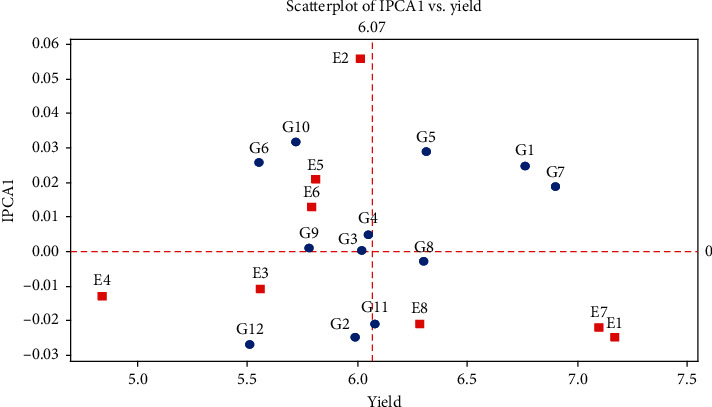
AMMI 1 biplot for main effects vs. IPCA1 in 12 hybrids of *Zea mays* L. from eight environments. G1: KSC703; G2: KSC260; G3: KSC705; G4: KSC400; G5: KSC706; G6: KSC704; G7: KSC707; G8: DC370; G9: SC647; G10: SC302; G11: SC604; G12: SC301. E1: Karaj Year 1; E2: Karaj Year 2; E3: Birjand Year 1; E4: Birjand Year 2; E5: Shiraz Year 1; E6: Shiraz Year 2; E7: Arak Year 1; E8: Arak Year2.

**Figure 2:**
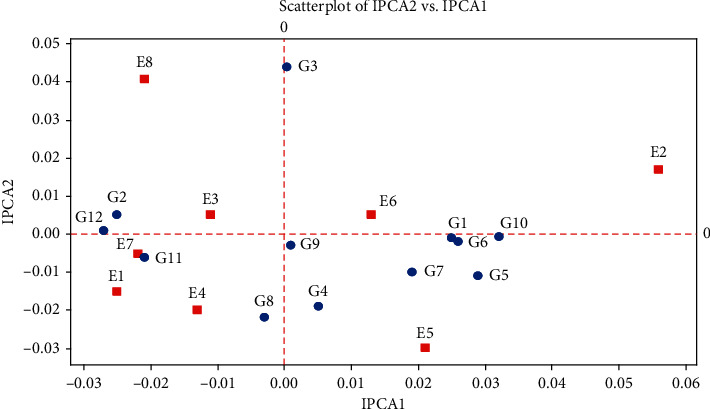
AMMI 2 biplot for the two main axes of interaction (IPCA 2 vs. IPCA 1) in 12 hybrids of *Zea mays* L. from eight environments. G1: KSC703; G2: KSC260; G3: KSC705; G4: KSC400; G5: KSC706; G6: KSC704; G7: KSC707; G8: DC370; G9: SC647; G10: SC302; G11: SC604; G12: SC301; E1: Karaj Year 1; E2: Karaj Year 2; E3: Birjand Year 1; E4: Birjand Year 2; E5: Shiraz Year 1.

The important goals of plant breeding are to select hybrids with wide adaptation in a series of condition that usually has good and stable performance. The most appropriate methods include identifying desirable cultivars with high productivity genetic potential and testing wide adaptability to most conditions by multi-condition experiments in target environments [27]. The present study determined the stability and adaptability of maize hybrids. Due to the significant combined analysis, the grain yield stability could be examined and hybrids with specific adaptability to each environment and hybrids with general adaptability to all environments can be measured. The significance of genotype × environment interaction indicated that environments could classify GEI [28, 29]. The SC647 genotype with the lowest AMMI stability value (0.001) was identified as the most stable genotype among 12 genotypes. The highest ASV related to the SC302 genotype (0.072). The sum of the yield and stability rankings (YSI) ranked KSC707 as the genotype that combined high yield with stability. The KSC706 genotype though high yielding was unstable because of its low rank according to the YSI. The two genotypes KSC400 and DC370 can be considered high yielding and stable (Table 4). Therefore, genotypes that have been considered stable genotypes based on ASV and IPCA parameters tend to have a lower average yield [30]. Using stability parameters on data obtained from four regions in the first crop year, KSC260, KSC705, SC647, and SC301 hybrids showed the highest value of the parameters. Also, KSC703, KSC707, and DC370 genotypes identified as the most unstable hybrids due to their high statistic levels. Based on the analysis of the four tested regions in the first crop year, the second crop year, and the mean of two crop years, and considering all the calculated parameters, SC647 and KSC705 genotypes identified as the most stable and KSC707 and KSC706 genotypes as the most unstable genotypes in case of grain yield. AMMI can have several models: AMMI0, which estimates the main additive effect of genotypes and environments and does not include any major axis (IPCA); AMMI1, which combines AMNI0 genotype additive effects with environmental interactions estimated and combines from the first major axis (IPCA 1); and AMMI2 and others up to the full model with all IPCA axes. [31]. According to the AMMI1 graph, which explained 73.4% of the total squares of the data variance, the environmental share was 38.36%, the genotype share was 12.01%, and the first component share was 23%. Accordingly, a genotype close to the mean axis and on the positive axis is more stable. KSC400, KSC705, and SC647 genotypes were more stable in comparison with other genotypes. KSC706, KSC703, and KSC707 genotypes are the most unstable, low-yielding genotypes, and the DC370 genotype, the most stable, high-yielding genotype. Environmental stability is essential for demonstrating the reliability of genotype ordering in a given environment about the rating for the environments in question [32]. According to the AMMI2 graph, 68.1% of the data variance explained 46.4% of the first component and 21.7% to the second component. Considering this graph, SC604 and SC302 genotypes in E7 and E1 environments; DC370 genotype in E4 environment; KSC400, KSC707, and KSC706 genotypes in E5 environment; and KSC260 and SC301 genotypes in E3 environment benefited from a desirable yield. Regarding the AMMI2 graph, the SC647 genotype was more stable than other genotypes, but unlike the AMMI1 graph with more stable KSC705 and KSC400 genotypes, these genotypes identified as unstable (Figures 1 and 2).

## 5. Conclusion

Regarding the AMMI model, the results of the analysis of variance indicated significant genotype × environment interaction. E2 environment selected as the most suitable environment for screening the genotypes. Based on the results of the data analysis of the four tested regions in the first crop year, the second crop year, and the mean of two crop years, and considering all the calculated parameters, SC647 and KSC705 genotypes were identified as the most stable and KSC707 and KSC706 genotypes as the most unstable genotypes in case of grain yield.

## Figures and Tables

**Table 1:** Annual rainfall mean, codes, and geographical parameters for different environments.

Location code		Location	Longitude	Latitude	Elevation AMSL (m)	Average rainfall (mm)
Year 1	Year 2					
E1	E2	Karaj	51.00	35.48	1321	295
E3	E4	Birjand	59.19	32.53	1420	171
E5	E6	Shiraz	52.32	29.36	1670	337.8
E7	E8	Arak	49.4	34.00	1708	341.7

**Table 2:** Names and codes of maize varieties studied in the experiment.

Genotype no.	Genotype	Means
G1	KSC 703	6.76 ab
G2	KSC 260	5.99 bc
G3	KSC 705	6.02 bc
G4	KSC 400	6.05 bc
G5	KSC 706	6.3 abc
G6	KSC 704	5.55 c
G7	KSC 707	6.9 a
G8	SC 307	6.3 abc
G9	SC 647	5.78 c
G10	SC 302	5.72 c
G11	SC 604	6.08 bc
G12	SC 301	5.51 c

**Table 3:** Combined analysis of variance for maize hybrid in four environments and two years.

Source of variation	DF	SS	MS
Local	3	110.1	36.7*∗*
Year	1	35.2	35.2*∗*
Year × Local	3	13.02	4.34*∗*
Error 1	16	46.4	2.9
Genotype	11	49.5	4.5*∗*
Genotype × Local	33	84.94	2.5*∗*
Genotype × Year	11	32.7	2.9*∗∗*
Genotype × Year × Local	33	87.18	2.6*∗*
Error 2	176	293.92	1.67

Symbols *∗*, *∗∗*, and ns denote significance at 5%, significance at 1%, and not significant, respectively.

**Table 4:** Ranking of 12 hybrids of maize and 8 environments based on the AMMI stability value (ASV).

		Mean (t/ha)	Rank (A)	IPCA 1	IPCA 2	ASV	ASV rank (B)	YSI (*A* + *B*)	YSI rank
Genotypes	G1	6.76	2	0.025	−0.001	0.056	8	10	4
	G2	5.99	8	−0.025	0.005	0.055	7	15	9
	G3	6.02	7	0.0004	0.044	0.044	5	12	7
	G4	6.05	6	0.005	−0.019	0.022	2	8	3
	G5	6.31	3	0.029	−0.011	0.066	11	14	8
	G6	5.55	11	0.026	−0.002	0.058	9	20	10
	G7	6.9	1	0.019	−0.010	0.043	4	5	1
	G8	6.3	4	−0.003	−0.022	0.023	3	7	2
	G9	5.78	9	0.001	−0.003	0.005	1	10	5
	G10	5.72	10	0.032	−0.0007	0.072	12	22	11
	G11	6.08	5	−0.021	−0.006	0.047	6	11	6
	G12	5.51	12	−0.027	0.001	0.060	10	22	12

Environments	E1	7.17	1	−0.022	−0.005	0.049	5	6	1
	E2	6.01	4	0.056	0.017	0.127	8	12	8
	E3	5.56	7	−0.011	0.005	0.025	1	8	4
	E4	4.84	8	−0.013	−0.02	0.035	3	11	7
	E5	5.81	5	0.021	−0.03	0.055	6	11	6
	E6	5.79	6	0.013	0.005	0.029	2	8	3
	E7	7.10	2	−0.022	−0.005	0.049	4	6	2
	E8	6.28	3	−0.021	0.041	0.062	7	10	5

G1: KSC703; G2: KSC260; G3: KSC705; G4: KSC400; G5: KSC706; G6: KSC704; G7: KSC707; G8: DC370; G9: SC647; G10: SC302; G11: SC604; G12: SC301. E1: Karaj Year 1; E2: Karaj Year 2; E3: Birjand Year 1; E4: Birjand Year 2; E5: Shiraz Year 1; E6: Shiraz Year 2; E7: Arak Year 1; E8: Arak Year 2.

**Table 5:** Mean gain yield and 8 estimates of parametric stability measures for maize yield across 4 environments in 2 years.

	Genotype	Mean	*S* ^2^ *X*_i_	CV	*W* ^2^	SH	*b*_*i*_	*S*_di_^2^	*Ri* ^2^
Year 1	G1	6.33	0.34	9.33	8.32	3.23	−0.06	0.51	0.02
	G2	5.96	1.90	23.16	3.79	1.57	0.64	1.49	0.47
	G3	5.97	1.54	20.81	1.24	0.28	0.76	0.44	0.8
	G4	7.01	3.81	27.48	2.18	0.67	1.21	0.93	0.83
	G5	6.97	1.32	16.53	1.02	0.19	0.72	0.27	0.86
	G6	6.18	1.08	16.79	1.09	0.22	0.66	0.18	0.88
	G7	6.95	1.42	9.31	8.04	3.11	−0.02	0.62	0.003
	G8	6.27	1.07	16.51	7.8	3.02	0.14	1.54	0.04
	G9	6.20	0.08	14.48	1.43	0.36	0.57	0.14	0.88
	G10	6.32	2.85	26.7	5.86	2.21	0.7	2.65	0.37
	G11	6.51	0.45	10.37	6.1	2.54	0.13	0.68	0.08
	G12	6.38	1.75	20.76	2.99	1.24	0.67	2.63	0.55

Year 2	G1	6.84	3.05	25.5	6.34	2.36	0.83	3.12	0.31
	G2	5.74	1.23	19.3	2.65	1.1	0.62	1.04	0.43
	G3	6.25	3.66	30.6	2.76	0.87	1.49	0.86	0.84
	G4	5.53	1.13	19.29	7.33	2.77	0.02	1.7	0.007
	G5	5.85	2.98	29.5	13.59	5.38	−0.06	4.47	0.001
	G6	5.21	1.41	0.03	3.12	0.23	3.33	8.65	28.19
	G7	6.76	0.63	0.11	0.67	0.2	1.37	3.96	10.54
	G8	6.14	−0.13	0.003	1.21	0.04	2.3	6.19	14.66
	G9	5.58	0.66	0.87	0.03	0.35	0.27	1.80	7.88
	G10	4.90	0.35	0.46	0.35	0.38	0.67	2.27	13.55
	G11	5.35	0.38	0.34	1.75	0.54	1.32	3.18	20.22
	G12	4.59	1.05	0.77	1.34	0.71	0.39	0.95	20.61

Average of two years	G1	6.58	1.03	15.44	4.97	1.9	0.31	1.29	0.16
	G2	5.85	0.62	13.53	1.12	0.46	0.57	0.1	0.89
	G3	6.11	2.06	23.52	0.67	0.11	1.04	0.33	0.89
	G4	6.31	1.29	17.99	2.29	0.79	0.65	0.84	0.56
	G5	6.41	1.29	17.74	4.46	1.69	0.44	1.44	0.25
	G6	6.7	1.01	17.65	3.02	1.09	0.5	0.88	0.41
	G7	6.86	0.24	7.18	4.42	1.67	0.13	0.31	0.13
	G8	6.21	0.46	10.97	5.7	2.22	0.07	0.68	0.01
	G9	5.89	0.44	11.33	1.48	0.45	0.48	0.07	0.89
	G10	5.61	0.85	16.43	2.68	0.95	0.48	0.67	0.47
	G11	5.93	0.41	10.82	3.07	1.28	0.31	0.61	0.41
	G12	5.48	0.83	16.6	1.17	0.49	0.62	1.24	0.8

G1: KSC703; G2: KSC260; G3: KSC705; G4: KSC400; G5: KSC706; G6: KSC704; G7: KSC707; G8: DC370; G9: SC647; G10: SC302; G11: SC604; G12: SC301.

## Data Availability

The data used to support the findings of this study are available from the corresponding author upon request.

## References

[1] http://www.fao.org/faostat/en/#home

[2] Alwala S., Kwolek T., McPherson M., Pellow J., Meyer D. (2010). A comprehensive comparison between Eberhart and Russell joint regression and GGE biplot analyses to identify stable and high yielding maize hybrids. *Field Crops Research*.

[3] Moghaddam A., Raza A., Vollmann J. (2013). Carbon isotope discrimination and water use efficiency relationships of alfalfa genotypes under irrigated and rain-fed organic farming. *European Journal of Agronomy*.

[4] Mousavi S. M. N., Kith K., Nagy J. (2019). Effect of interaction between traits of different genotype maize in six fertilizer level by GGE biplot analysis in Hungary. *Progress in Agricultural Engineering Sciences*.

[5] Miah M., Ahmed S., Uddin M. (2016). Assessment of yield stability of maize inbred lines in multi-environment trials. *Bangladesh Journal of Scientific and Industrial Research*.

[6] Rakshit S., Ganapathy K. N., Gomashe S. S. (2012). GGE biplot analysis to evaluate genotype, environment and their interactions in sorghum multi-location data. *Euphytica*.

[7] Yan W., Pageau D., Frégeau-Reid J., Durand J. (2011). Assessing the representativeness and repeatability of test locations for genotype evaluation. *Crop Science*.

[8] Gupta P., Dhawan S. S., Lal R. K. (2015). Adaptability and stability based differentiation and selection in aromatic grasses (Cymbopogon species) germplasm. *Industrial Crops and Products*.

[9] Przystalski M., Osman A., Thiemt E. M. (2008). Comparing the performance of cereal varieties in organic and non-organic cropping systems in different European countries. *Euphytica*.

[10] Farshadfar E., Farshadfar M., Sutka J. (2001). Combining ability analysis of drought tolerance in wheat over different water regimes. *Acta Agronomica Hungarica*.

[11] Ssemakula G. N., Dixon A. (2007). Genotype × environment interaction, stability and agronomic performance of carotenoid-rich cassava clones. *Scientific Research and Essays*.

[12] Bose L. K., Jambhulkar N. N., Pande K., Singh O. N. (2014). Use of AMMI and other stability statistics in the simultaneous selection of rice genotypes for yield and stability under direct-seeded conditions. *Chilean Journal of Agricultural Research*.

[13] Giridhar K., Kumari S. S., Sarada C., Naidu L. (2016). Stability for seed yield in ajwain based on gentoype selection index. *Indian Journal of Agricultural Research*.

[14] Mousavi S. M. N., Nagy J. (2020). Evaluation of plant characteristics related to grain yield of FAO410 and FAO340 hybrids using regression models. *Cereal Research Communications*.

[15] Purchase J. L., Hatting H., Van Deventer C. S. (2000). Genotype × environment interaction of winter wheat (*Triticum aestivum* L.) in South Africa: II. Stability analysis of yield performance. *South African Journal of Plant and Soil*.

[16] Temesgen T., Keneni G., Sefera T., Jarso M. (2015). Yield stability and relationships among stability parameters in faba bean (*Vicia faba* L.) genotypes. *The Crop Journal*.

[17] Farshadfar E., Mahmodi N., Yaghotipoor A. (2011). AMMI stability value and simultaneous estimation of yield and yield stability in bread wheat (‘*Triticum aestivum*’L.). *Australian Journal of Crop Science*.

[18] Baraki F. (2014). AMMI analysis of genotype × environment (*G* × *E*) interaction and stability of sesame genotypes in Northern Ethiopia. Results of crop research. *Asian Journal of Plant Sciences*.

[19] Illés Á., Mousavi S. M. N., Bojtor C., Nagy J. (2020). The plant nutrition impact on the quality and quantity parameters of maize hybrids grain yield based on different statistical methods. *Cereal Research Communications*.

[20] Shahbazi E. (2019). Genotype selection and stability analysis for seed yield of Nigella sativa using parametric and non-parametric statistics. *Scientia Horticulturae*.

[21] Liu Y.-J., Wei B., Hu E.-L., Wu Y.-q., Huang Y.-b. (2011). Yield stability of maize hybrids evaluated in national maize cultivar regional trials in Southwestern China using parametric methods. *Agricultural Sciences in China*.

[22] Eberhart S. A., Russell W. A. (1966). Stability parameters for comparing varieties 1. *Crop Science*.

[23] Abate M. (2020). Genotype by environment interaction and yield stability analysis of open pollinated maize varieties using AMMI model in Afar Regional State, Ethiopia. *Journal of Plant Breeding and Crop Science*.

[24] Adjebeng-Danquah J., Manu-Aduening J., Gracen V. E., Asante I. K., Offei S. K. (2017). AMMI stability analysis and estimation of genetic parameters for growth and yield components in cassava in the forest and Guinea Savannah ecologies of Ghana. *International Journal of Agronomy*.

[25] Tumuhimbise R., Melis R., Shanahan P., Kawuki R. (2014). Genotype × environment interaction effects on early fresh storage root yield and related traits in cassava. *The Crop Journal*.

[26] Kandus M., Almorza D., Boggio Ronceros R., Salerno J. C. (2010). Statistical models for evaluating the genotype-environment interaction in maize (Zea mays L.). *Phyton-Revista Internacional de Botanica Experimental*.

[27] Tena E., Goshu F., Mohamad H., Tesfa M., Tesfaye D., Seife A. (2019). Genotype × environment interaction by AMMI and GGE-biplot analysis for sugar yield in three crop cycles of sugarcane (*Saccharum officinirum* L.) clones in Ethiopia. *Cogent Food & Agriculture*.

[28] Yan W., Hunt L. A., Sheng Q., Szlavnics Z. (2000). Cultivar evaluation and mega-environment investigation based on the GGE biplot. *Crop Science*.

[29] Ma B. L., Yan W., Dwyer L. M. (2004). Graphic analysis of genotype, environment, nitrogen fertilizer, and their interactions on spring wheat yield. *Agronomy Journal*.

[30] Bashir E. M. A., Ali A. M., Ali A. M., Ismail M. I., Parzies H. K., Haussmann B. I. G. (2014). Patterns of pearl millet genotype-by-environment interaction for yield performance and grain iron (Fe) and zinc (Zn) concentrations in Sudan. *Field Crops Research*.

[31] Nowosad K., Liersch A., Popławska W., Bocianowski J. (2016). Genotype by environment interaction for seed yield in rapeseed (*Brassica napus* L.) using additive main effects and multiplicative interaction model. *Euphytica*.

[32] Rocha M. d. M., Freire Filho F. R., Ribeiro V. Q. (2007). Yield adaptability and stability of semi-erect cowpea genotypes in the Brazil Northeast Region. *Pesquisa Agropecuária Brasileira*.

